# Supplementary material for: Novel Therapeutic Strategies Exploiting the Unique Properties of Neuroendocrine Neoplasms
Source: Cancers (Basel). 2023 Oct 12;15(20):4960. doi: 10.3390/cancers15204960 (PMC10605125; doi:10.3390/cancers15204960)
Supplement: Supplementary file 1 [file cancers-15-04960-s001.zip › cancers-2596639-supplementary.pdf]

Supplementary Figure and Table Legends

Supplementary Table S1. Neuroendocrine cell lines subjected to library screen. Characteristics of the three neuroendocrine cells used in the initial library screening.

Supplementary Table S2. GSEA (Gene Set Enrichment Analysis) results for the contrast NET vs. other. The 50 Hallmark gene sets from the MSigDB (Molecular Signature Database) human collection (https://www.gsea-msigdb.org/gsea/msigdb/human/genesets.jsp?collection=H) were used for the enrichment analysis. For details, please see Ref. 1.

Supplementary Figure S1. Ratification of HDAC inhibitors activity on neuroendocrine cancer cells. The sensitivity to the HDAC inhibitors, romidepsin, belinostat, pracinostat, givinostat, quisinostat, panobinostat and dacinostat, is ratified using a panel of 13 neuroendocrine cell lines. Activity is plotted as percent of control. Concentrations are Log [M].

Supplementary Figure S2. Differentially expressed NET genes based on Ingenuity Pathway Analysis. A) Supervised analysis of cell lines based on the expression of genes with a Log2 fold change absolute value >2 (Var>0.1, $p<0.05$) identified 557 differentially expressed genes between the insensitive and sensitive group among 4,293 genes annotated as of neuroendocrine origin. **B).** Differentially expressed medulla genes. Supervised analysis of cell lines based on the expression of genes with a Log2 fold change absolute value >1.5 (Var>0.1, $p<0.05$) identified 12 differentially expressed genes between the insensitive and sensitive group, utilizing a 30 gene set identified as encoding proteins expressed in the human adrenal medulla by The Human Protein Atlas.

Supplementary Figure S3. SLC52A3 and YAP1 RNA levels in neuroendocrine cancer cells. SLC52A3 and YAP1 RNA levels are expressed as Log2Fold. Kelly, NH6 and NCI-H82 cells show lower RNA levels when compared to GCIY, OE-19, SNU-119, NCI-H2342 and UOK-269 cells.

Supplementary Figure S4. Riboflavin deficiency plays a role in Kelly and NH6 sensitivity to NAMPT inhibitors. A) SLC52A3 protein expression analysis in sensitive and resistant cells. **B)** Riboflavin addition protects Kelly and NH6 from NAMPTs inhibitor cytotoxicity. Columns represent percent of dead cells. Bars show average standard deviation of three independent experiments.

Supplementary Figure S5. Reduced ATP in SNU-119 and OE-19 cell lines by NAMPT inhibitors plus CA3. Levels of ATP production via mitochondrial respiration and glycolysis in SNU-119 and OE-19 cell lines exposed to GMX1778 (10nM) and STF1188804 (20nM) plus CA3(0.5 μM) for 24h. ATP production is measured as pmol/min/cell x 1000. ATP production is expressed as 100% in untreated cells (control). Bars represent standard deviations of three independent results.

References for Supplementary Figures and Tables

1. Gene set enrichment analysis: A knowledge-based approach for interpreting genome-wide expression profiles. Subramanian, A., Tamayo, P., Mootha, V. K., Mukherjee, S., Ebert, B. L., Gillette, M. A., Paulovich, A., Pomeroy, S. L., Golub, T. R., Lander, E. S. & Mesirov, J. P. Proc. Natl. Acad. Sci. USA (2005) 102:15545-50.

Supplementary Table S1. Neuroendocrine cell lines subjected to library screen.

	**NCI-H69 [H69] (ATCC® HTB119™)**	**DMS 79 (ATCC® CRL2049™)**	**NCIH727 [H727] (ATCC® CRL5815™)**
Organism	Homo sapiens	Homo sapiens	Homo sapiens
Tissue	Lung	Lung/pleural fluid	Lung, bronchus
Disease	Carcinoma; small cell lung cancer	Carcinoma; small cell lung cancer	Carcinoid
Age	55 years	65 years	66 years
Gender	Male	N/A	Female
Morphology	Floating aggregates	Floating aggregates	Epithelial
Growth Properties	Suspension, multicell aggregates	Suspension, multicell aggregates	Adherent
KRAS	Negative	Negative	Positive (G12V, one allele)

Supplementary Table S1, Safari et al

Supplementary Table S2

HALLMARK Name	Size	Matches	ES	abs(ES)	NES	p	q
GLYCOLYSIS	200	147	-0.43215	0.432152	-1.52533	0.0000	0.142742
APOPTOSIS	161	128	-0.43921	0.439208	-1.54374	0.0000	0.140372
APICAL JUNCTION	200	177	-0.40186	0.401862	-1.55682	0.0000	0.13301
IL6 JAK STAT3 SIGNALING	87	73	-0.56721	0.567205	-1.58883	0.0000	0.125188
COAGULATION	138	118	-0.48671	0.486713	-1.62693	0.0000	0.095041
TNFA SIGNALING VIA NFKB	200	178	-0.59339	0.593393	-1.62731	0.0000	0.10056
ANDROGEN RESPONSE	100	76	-0.47517	0.475174	-1.63339	0.0000	0.112286
ESTROGEN RESPONSE LATE	200	180	-0.52343	0.523428	-1.67717	0.0000	0.088398
P53 PATHWAY	200	161	-0.53431	0.53431	-1.68543	0.0000	0.091975
CHOLESTEROL HOMEOSTASIS	74	58	-0.59071	0.590708	-1.72817	0.0000	0.089293
INFLAMMATORY RESPONSE	200	174	-0.49973	0.499734	-1.74054	0.0000	0.129532
ESTROGEN RESPONSE EARLY	200	186	-0.56064	0.560636	-1.8528	0.0000	0.093508
APICAL SURFACE	44	41	-0.42136	0.42136	-1.47537	0.0207	0.154288
PROTEIN SECRETION	96	50	-0.54142	0.541422	-1.49476	0.0236	0.144821
IL2 STAT5 SIGNALING	199	176	-0.44637	0.44637	-1.54013	0.0336	0.1397
TGF BETA SIGNALING	54	39	-0.65942	0.659417	-1.73507	0.0361	0.09964
PI3K AKT MTOR SIGNALING	105	72	-0.39226	0.392261	-1.43808	0.0365	0.1689
COMPLEMENT	200	168	-0.4397	0.439695	-1.51489	0.0398	0.141199
INTERFERON ALPHA RESPONSE	97	80	-0.65863	0.658631	-1.62829	0.0398	0.104239
INTERFERON GAMMA RESPONSE	200	163	-0.58931	0.589307	-1.64293	0.0421	0.122634
PANCREAS BETA CELLS	40	35	0.597306	0.597306	1.9042	0.0515	0.072785
SPERMATOGENESIS	135	90	0.469632	0.469632	1.677	0.0524	0.119635
UV RESPONSE DN	144	122	-0.35999	0.359993	-1.28181	0.0538	0.251547
REACTIVE OXYGEN SPECIES PATHWAY	49	36	-0.46741	0.467405	-1.39445	0.0622	0.177052
ADIPOGENESIS	200	120	-0.4583	0.458299	-1.53683	0.0646	0.14122
XENOBIOTIC_METABOLISM	200	161	-0.39881	0.398808	-1.47295	0.0690	0.148839
HYPOXIA	200	177	-0.36582	0.365815	-1.37976	0.0722	0.183553
HEME METABOLISM	200	143	-0.35937	0.359365	-1.31541	0.0759	0.221813
BILE ACID METABOLISM	112	93	-0.36758	0.367584	-1.39738	0.0792	0.179413
FATTY ACID METABOLISM	158	105	-0.38542	0.385423	-1.3223	0.0861	0.226026
E2F TARGETS	200	96	0.57725	0.57725	1.56721	0.0915	0.127444
OXIDATIVE PHOSPHORYLATION	200	73	-0.47192	0.471921	-1.40128	0.0996	0.184078
HEDGEHOG SIGNALING	36	34	0.476058	0.476058	1.53039	0.1005	0.119426
G2M CHECKPOINT	200	95	0.543436	0.543436	1.47765	0.1029	0.129089
ALLOGRAFT REJECTION	200	153	-0.37956	0.379556	-1.42843	0.1204	0.169021
NOTCH SIGNALING	32	25	-0.46752	0.467518	-1.32748	0.1217	0.22616
WNT BETA CATENIN SIGNALING	42	33	0.335298	0.335298	1.27756	0.1355	0.309127
ANGIOGENESIS	36	32	-0.41284	0.41284	-1.27191	0.1883	0.253411
UV RESPONSE UP	158	112	-0.31816	0.318164	-1.14175	0.2171	0.371803
PEROXISOME	104	73	-0.3237	0.323696	-1.11668	0.2350	0.373419
UNFOLDED PROTEIN RESPONSE	113	57	-0.30433	0.304332	-1.05944	0.2865	0.427046
MTORC1 SIGNALING	200	126	-0.37347	0.373474	-1.12176	0.2986	0.378496
MYOGENESIS	200	174	-0.27313	0.273127	-1.03024	0.3630	0.442651
EPITHELIAL MESENCHYMAL TRANSITION	200	192	-0.31124	0.311236	-1.04132	0.3893	0.435515
KRAS SIGNALING UP	200	179	-0.25833	0.258329	-1.00109	0.4435	0.465614
MYC TARGETS V2	58	27	0.422129	0.422129	0.958635	0.5633	0.711597
MYC TARGETS V1	200	57	0.331726	0.331726	0.875717	0.5869	0.678723
MITOTIC SPINDLE	199	123	0.253314	0.253314	0.926956	0.7265	0.680791
KRAS SIGNALING DN	200	173	0.200766	0.200766	0.842843	0.7454	0.650799
DNA REPAIR	150	61	-0.20602	0.206023	-0.67157	0.9427	0.874454

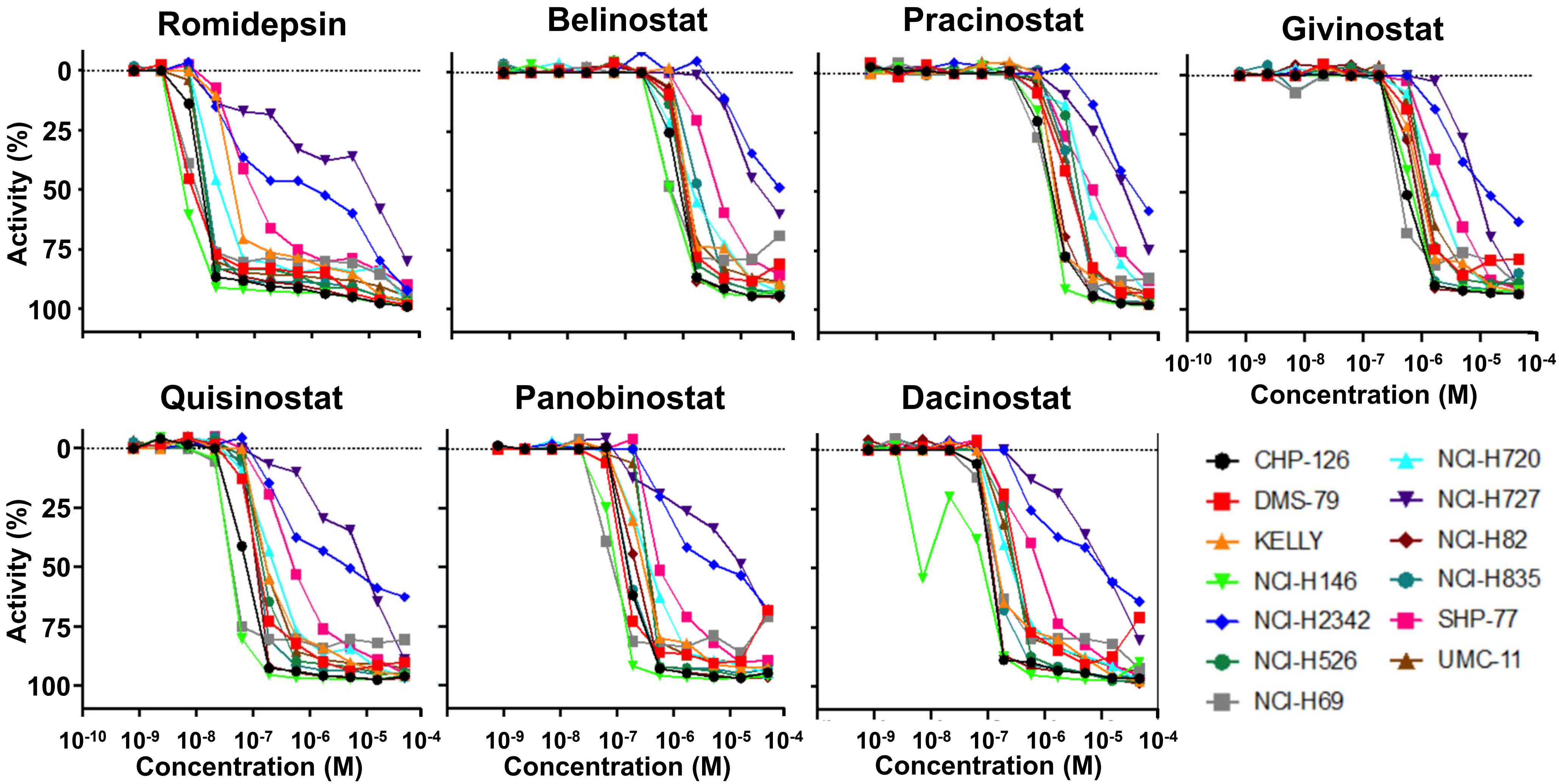

Supplementary Figure S1, Safari et al

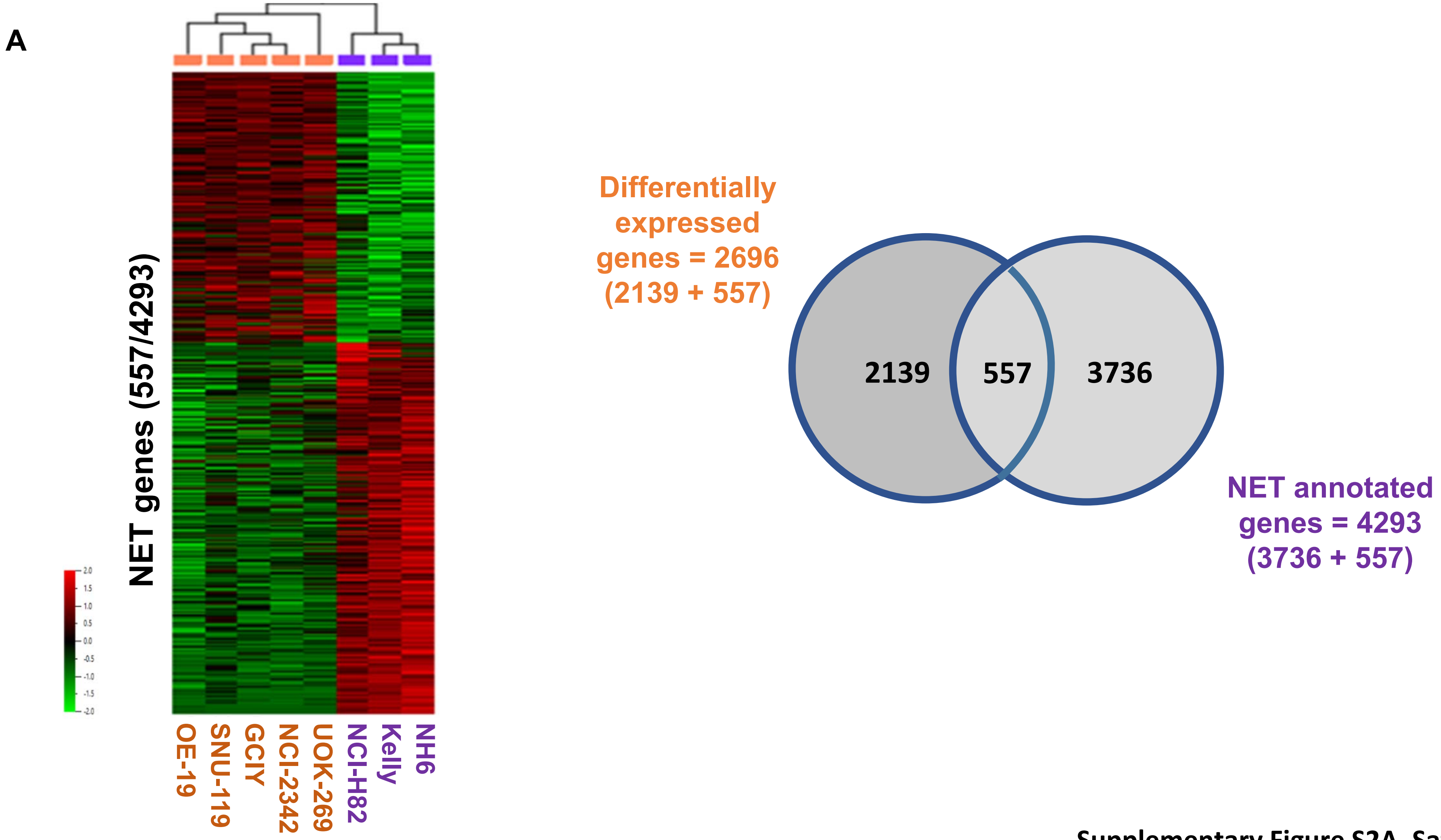

Supplementary Figure S2A, Safari et al

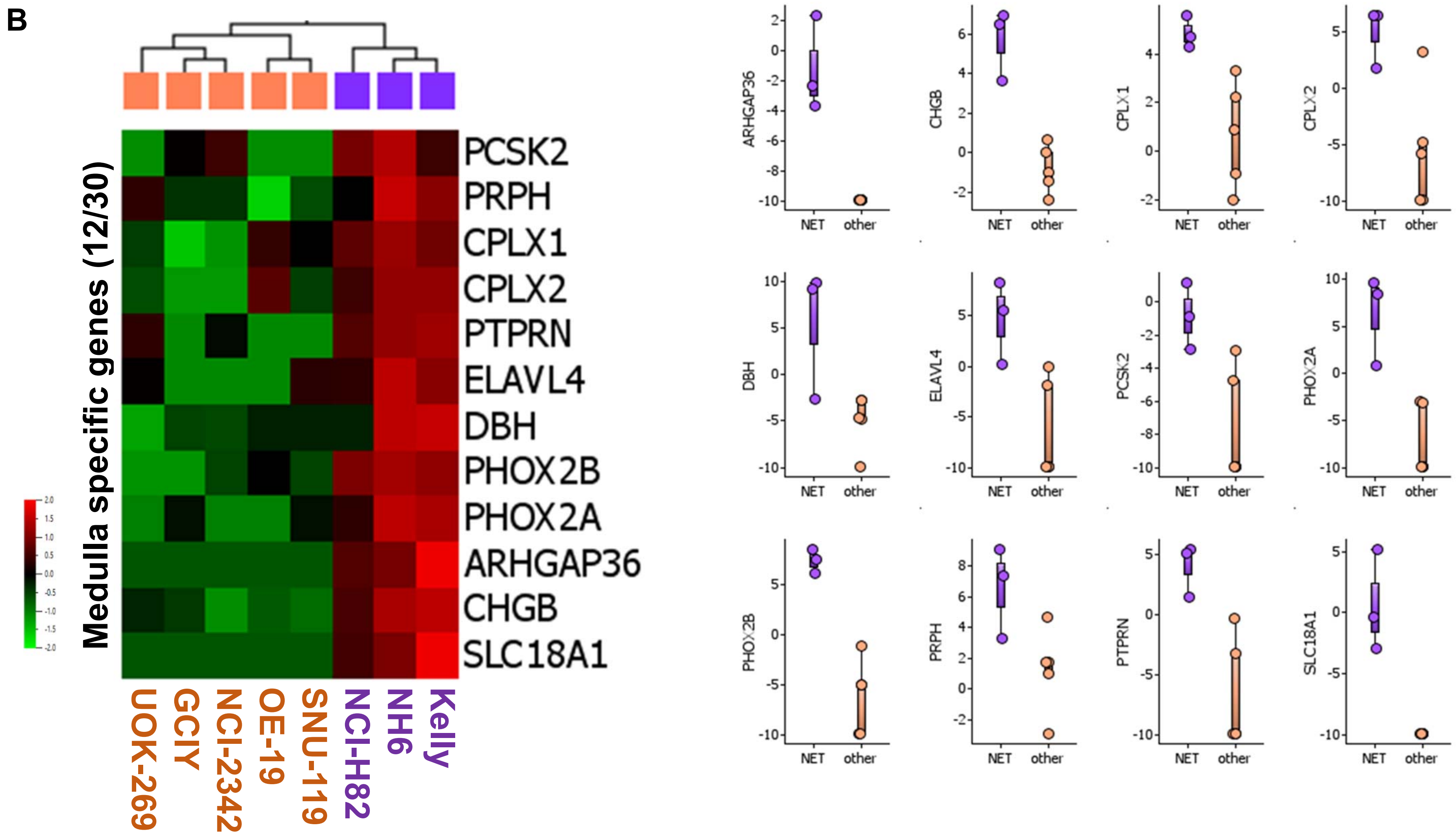

Supplementary Figure S2B, Safari et al

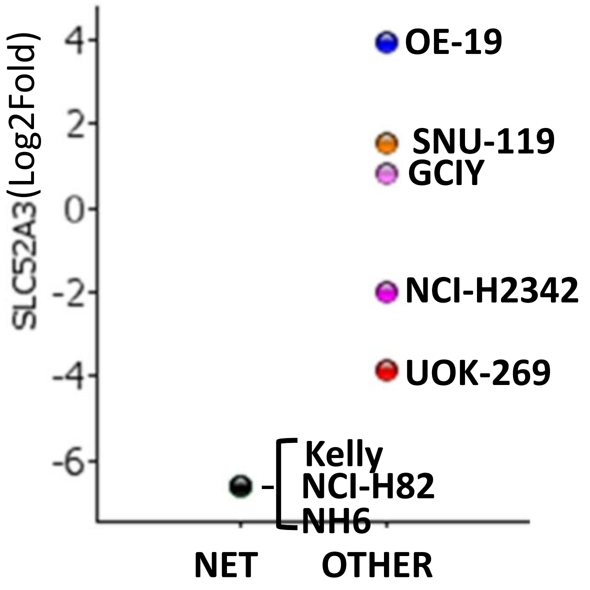

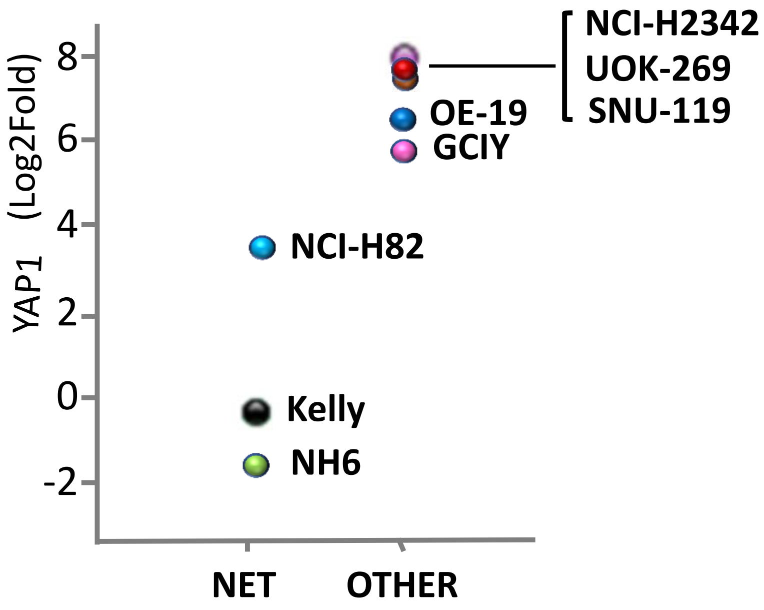

Supplementary Figure S3, Safari et al

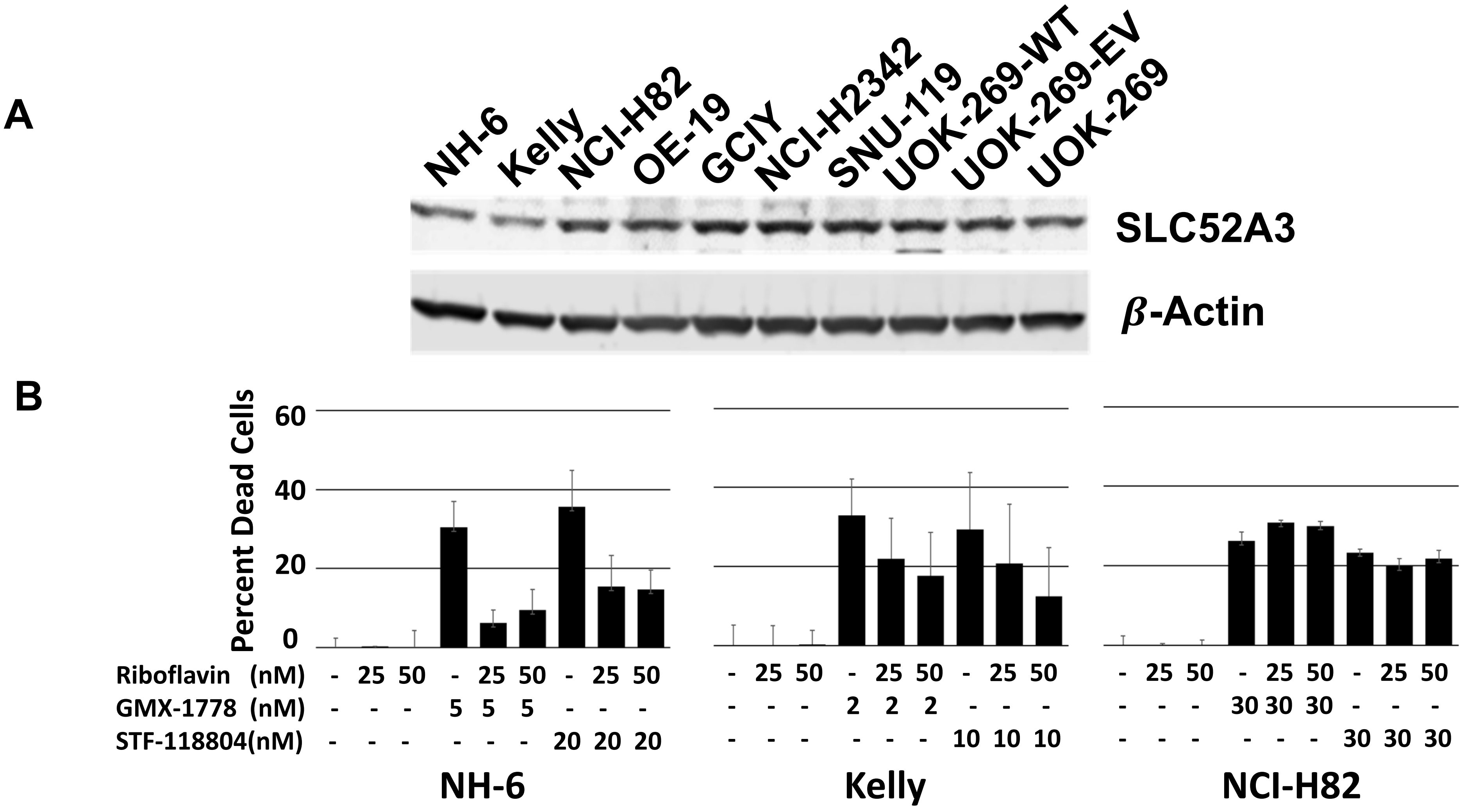

Supplementary Figure S4, Safari et al

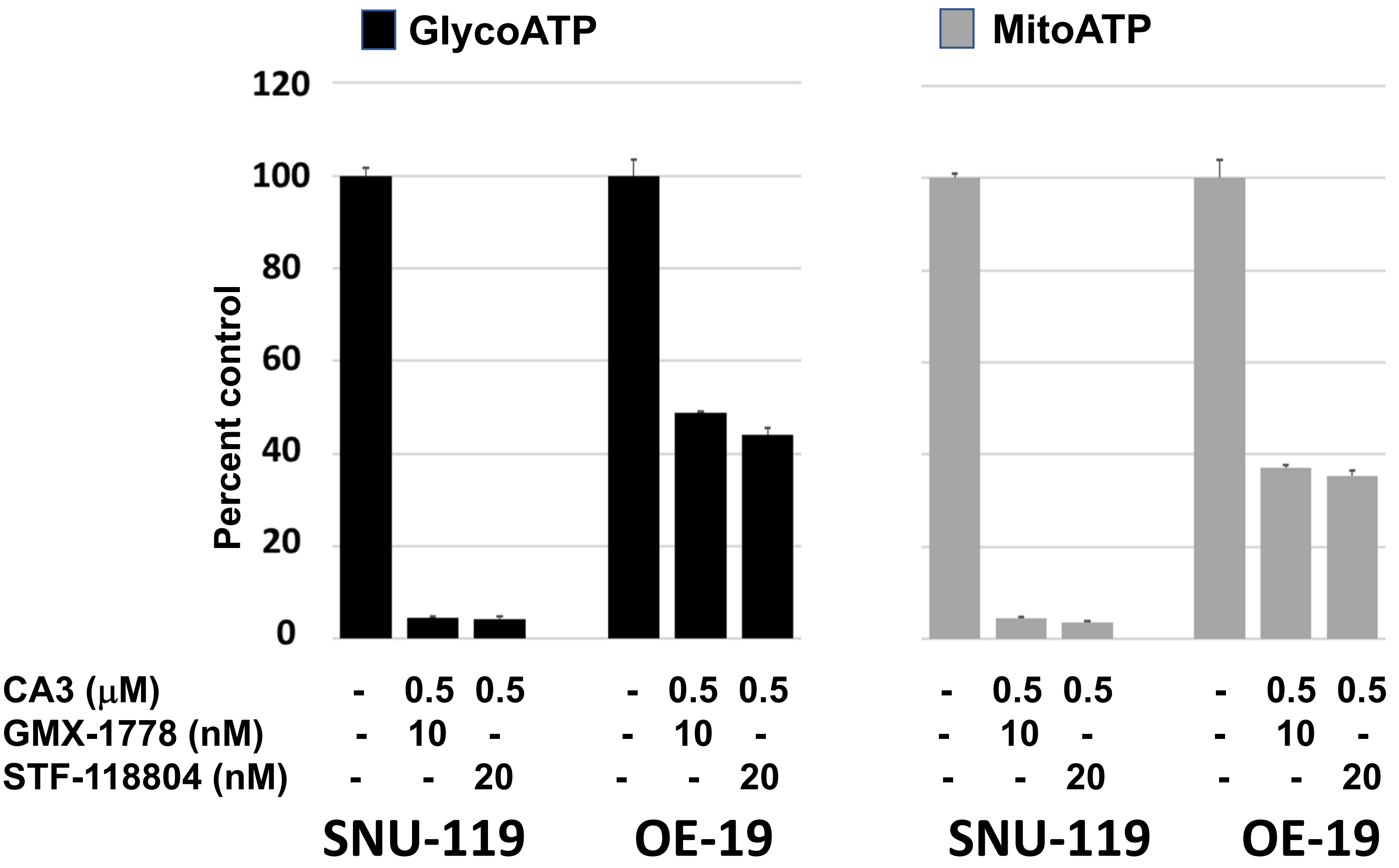

Supplementary Figure S5. Safari et al

High-throughput screening

For small molecule library screening, cells were suspended in appropriate cell culture medium (supplemented with 10% FBS, 100-units/L penicillin-streptomycin and 1% glutamine), and then dispensed to white solid bottom 1536-well tissue culture plates (Greiner Bio-one) at a density of 1,000 cells in 5 µL of media (MultiDrop Combi, Thermofisher Scientific, MA). After incubating at 37°C, 5% CO_2 overnight, 20 nL of DMSO-solubilized compounds from compound plates were transferred (Wako Automation, San Diego, CA) to the cell assay plate from column 5 to column 48. The controls were added in columns 1 to 4 of each assay plate. Columns 1 and 2 were DMSO control. After 48 hours of incubation, 3 µL of Cell-TiterGlo reagent (Promega, WI) was added to each well. Following a 10 min incubation, relative luminescence units (RLU) was read in the Viewlux microplate reader (PerkinElmer, MA).

Analysis of compound concentration–response data was performed as previously described [13]. Concentration–response titration points for each compound were fitted to a four-parameter Hill equation [1] yielding concentrations of half-maximal activity (IC_{50}) and maximal response (efficacy) values. Compounds were designated as Class 1–4 according to the type of concentration–response curve observed [2, 3]. Curve classes are heuristic measures of data confidence, classifying concentration–responses based on efficacy, the number of data points observed above background activity, and the quality of fit. Compounds with curve classes 1.1, 1.2, 2.1, 2.2 were considered active. Class 4 compounds were considered inactive. Compounds with other curve classes were deemed inconclusive. Active compounds were cherrypicked for follow up experiments.

Clustering of compounds by activity outcomes.

Compounds were clustered hierarchically using TIBCO Spotfire 6.0.0 (Tibco Software Inc., Cambridge, MA. https://spotfire.tibco.com/) based on their activity outcomes from the primary or follow up screens across different methyltransferases. Compound's AUC (Area Under the Curve) calculated based on the qHTS data analysis and curve fittings were utilized for clustering. In the heatmap, darker red indicates compounds that are more potent and efficacious, i.e., high-quality actives, and lighter red indicates less potent and efficacious compounds. If a compound didn't show any activity in an assay, it was highlighted as white in the heatmap.

Evaluation of riboflavin protective role on cell viability.

Kelly, NH6 and NCI-H82 cells were cultured in growth medium supplemented with 10% inactivated fetal bovine serum. For the analysis of the protective role of riboflavin versus cytotoxicity, riboflavin was added at a final concentration of 25 and 50nM in absence or presence of GMX1778 (10nM) and STF11804 (20nM). After 48h culture, cells were harvested, and the viability was determined by using the annexin V fluorescein isothiocyanate (annexin V-FITC) Apoptosis Detection Kit (BD Biosciences, San Diego, CA) according to the manufacturer's instructions. Following treatment, annexin positive cells were quantitated by FACS analysis (Becton Dickinson, Ranklin Lakes, NJ) using FCS Express Cytometry Software (Tree Star, Inc, Ashland, OR). The percentage of annexin positive was calculated and the delta between treated and untreated control was determined.

1 HILL A. The possible effects of the aggregation of the molecules of haemoglobin on its dissociation curves. *J Physiol* 1910; 14: iv-vii.

2 Inglese J, Auld DS, Jadhav A, Johnson RL, Simeonov A, Yasgar A *et al*. Quantitative high-throughput screening: a titration-based approach that efficiently identifies biological activities in large chemical libraries. *Proc Natl Acad Sci U S A* 2006; 103: 11473-11478.

3 Huang RL, Xia MH, Cho MH, Sakamuru S, Shinn P, Houck KA *et al*. Chemical Genomics Profiling of Environmental Chemical Modulation of Human Nuclear Receptors. *Environ Health Persp* 2011; 119: 1142-1148.
